# Comparing static and dynamic emotion recognition tests: Performance of healthy participants

**DOI:** 10.1371/journal.pone.0241297

**Published:** 2020-10-28

**Authors:** Sara Khosdelazad, Lieke S. Jorna, Skye McDonald, Sandra E. Rakers, Rients B. Huitema, Anne M. Buunk, Jacoba M. Spikman

**Affiliations:** 1 Department of Neuropsychology, University of Groningen, University Medical Centre Groningen, Groningen, The Netherlands; 2 School of Psychology, University of New South Wales, Sydney, Australia; National Institutes of Health, UNITED STATES

## Abstract

Facial expressions have a communicatory function and the ability to read them is a prerequisite for understanding feelings and thoughts of other individuals. Impairments in recognition of facial emotional expressions are frequently found in patients with neurological conditions (e.g. stroke, traumatic brain injury, frontotemporal dementia). Hence, a standard neuropsychological assessment should include measurement of emotion recognition. However, there is debate regarding which tests are most suitable. The current study evaluates and compares three different emotion recognition tests. 84 healthy participants were included and assessed with three tests, in varying order: a. Ekman 60 Faces Test (FEEST) b. Emotion Recognition Task (ERT) c. Emotion Evaluation Test (EET). The tests differ in type of stimuli from static photographs (FEEST) to more dynamic stimuli in the form of morphed photographs (ERT) to videos (EET). Comparing performances on the three tests, the lowest total scores (67.3% correct answers) were found for the ERT. Significant, but moderate correlations were found between the total scores of the three tests, but nearly all correlations between the same emotions across different tests were not significant. Furthermore, we found cross-over effects of the FEEST and EET to the ERT; participants attained higher total scores on the ERT when another emotion recognition test had been administered beforehand. Moreover, the ERT proved to be sensitive to the effects of age and education. The present findings indicate that despite some overlap, each emotion recognition test measures a unique part of the construct. The ERT seemed to be the most difficult test: performances were lowest and influenced by differences in age and education and it was the only test that showed a learning effect after practice with other tests. This highlights the importance of appropriate norms.

## Introduction

Social cognition is the ability to form representations of others’ mental states (i.e. feelings, experiences, beliefs, and intentions) in relation to oneself, and guide social behavior by using these representations [1]. A crucial aspect of social cognition is facial emotion recognition. Facial expressions have important communicatory functions and the ability to read them is a prerequisite for understanding feelings and thoughts of other individuals [2]. There is substantial evidence that incorrect recognition and misinterpretations of emotional facial expressions is associated with impairments in social functioning, such as diminished social competence, poor social communication and inappropriate interpersonal behavior [3, 4]. Impaired recognition of emotional facial expressions has been documented in various neurological patient groups, including traumatic brain injury (TBI, [5–7]), stroke [8–10], and various neurodegenerative disorders, such as Alzheimer’s disease (AD, [11, 12]), frontotemporal dementia (FTD, [13, 14]), and Parkinson’s disease (PD, [15, 16]). At present, measurements of social cognition (i.e. emotion recognition) are often not included in standard neuropsychological assessment [17]. Hence, while deficits in emotion recognition represent an important target for assessment and treatment in clinical settings, they are not routinely assessed. An important step forward to remedy this situation, is to know which instruments are most suitable to measure such deficits.

At present, a number of neuropsychological tests have been developed to assess facial emotion recognition. These tests, however, substantially differ in the way the emotional information is conveyed to the participant. Test stimuli can be static displays of posed emotional expressions (e.g. photographs) or more dynamic in the form of morphed photographs that starts neutral and change gradually, or videos in which visual emotional expressions, associated with vocal cues are provided within a context. These differences in modality and presentation may challenge emotion recognition abilities in quite different ways. Humphreys, Donnely, and Riddoch [18] were one of the first investigators to suggest that recognition of static and dynamic facial emotional stimuli are based upon two distinct processes. Their study demonstrated that patients with selective impairments in the ability to recognize static emotional expressions, were still able to correctly recognize dynamic emotional expressions, and vice versa. Hence, the process of recognizing static and dynamic facial emotional stimuli seems to rely on partially distinct neural networks [19, 20]. Dynamic images are found to elicit more activity in brain regions associated with the interpretation of social aspects and emotional processing than static images [21]. Hence, dynamic test stimuli may have a higher predictive value for everyday social functioning [22, 23]. However, because of their dynamic nature, it is also likely that such tests put higher demands on information processing capacities than static tests. Indeed, studies showed that cognitive impairments, in particular mental speed, attention, and working memory, affect recognition of emotional facial expressions as measured with dynamic tests [24–26]. Westerhof-Evers and colleagues [27] hypothesized that dynamic test stimuli activate general neuropsychological processes to a greater extent than static test stimuli. To date, there has been only one study that directly compared static and dynamic emotion recognition tests, and their relation to other neuropsychological functions. McDonalds & Saunders [28] presented emotional stimuli using four different media within the same test: audiovisual, audio only, dynamic visual only, and static visual only. They found that low information processing speed (but not working memory) predicted poor performance, to a similar degree, across tasks with the exception of the audiovisual condition. This finding was limited to experimental manipulations of a small number of items in a single test (The Awareness of Social Inference Test). In general, there is evidence that the processing of emotions expressed through separate sensory channels (e.g. the voice and the face) entail different neural systems [29]. For example, evidence shows that there exists a dissociation between the ability to recognize emotions in voice and face in people with brain lesions [30]. It is yet to be demonstrated whether different, established tests of emotion recognition that vary in terms of (1) static vs. dynamic and (2) visual only vs. audiovisual presentations rely differently on cognitive skills.

Furthermore, it is important to take the effects of demographical factors, such as age, sex, and educational level into account. Research has shown that with advancing age the ability to correctly recognize facial emotions declines [31–33]. Moreover, ageing seems to be accompanied by the decline of various cognitive abilities that are relevant to performance on dynamic emotion recognition tests [34–36]. Also, the literature is inconsistent regarding sex differences in facial emotion recognition. Whereas some studies generally report a female advantage over males [37–39], others have not [40, 41]. Lastly, higher education seems to be correlated to better emotion recognition performance on both static [42, 43] and dynamic tests [27, 44].

This study aimed to compare performance on three different emotion recognition tests in a sample of healthy subjects. We used (a) the Ekman 60 Faces Test, a subtest of the Facial Expression of Emotion Stimuli and Test (FEEST) that makes use of static photographs [45], (b) the Emotion Recognition Task (ERT), which consists of morphed facial stimuli that gradually increase in intensity [46], and (c) The Emotion Evaluation Task (EET), a subtest of The Awareness of Social Inference Test (TASIT), which comprises audiovisual portrayals of emotion [47]. Although the three tests differ in stimuli type, they all make use of the same six basic emotions: anger, fear, disgust, happiness, sadness, and surprise [48].

Second, we aimed to examine the extent to which demographic variables (i.e. gender, age, educational level) and neuropsychological functions (i.e. working memory, attention, information processing speed) influenced the ability to correctly recognize facial emotions, and whether this differed between the three tests. Lastly, we investigated the extent to which each test would be susceptible to practice effects. We expect that our findings will contribute to a better understanding of the usefulness of these tests in clinical practice.

## Methods

### Participants and procedure

Eighty-four healthy participants (39 male, 45 female) with a mean age of 30.77 years (SD = 13.73, range 18–61) were included in this study. Participants for this study were recruited through convenience sampling. Exclusion criteria were age younger than 18 years and the presence or history of serious neurological or psychiatric disorders (including depression and anxiety). Educational level was scored according to a Dutch classification system [49]. Our sample consisted of participants with the three following educational categories: finished average-level secondary education (21.4%), finished high level secondary education (46.5%), and finished university degree (32.1%). These categories represent almost 80% of the Dutch population [50]. Three protocols of the test battery were used, each with a different order of the three emotion recognition tests (version 1: FEEST-EET-ERT; version 2: EET-ERT- FEEST; version 3: ERT-FEEST-EET). 26 participants (31%) completed version 1 of the emotion recognition test battery, 30 completed version 2 (35.7%), and 28 (33.3%) completed version 3. Furthermore, two other tests were added to measure neuropsychological functions.

Participants were tested individually at their home or (if not feasible) at the University Medical Centre Groningen, the Netherlands. The administration time of the complete test battery was approximately 1.5 hours. Ethical approval for this study was given by the Ethical Committee of Psychology (ECP) of the University of Groningen. All participants were treated in accordance with the Helsinki Declaration and gave written informed consent prior to testing.

### Measurement instruments

#### Emotion recognition

*The Ekman 60 faces test of the Facial Expressions of Emotion Stimuli and Tests* (FEEST) [45]. Participants are shown sixty photographs of faces, depicting the following six basic emotions: anger, disgust, fear, happiness, sadness, and surprise (ten of each). Each photograph is presented for 5 seconds on a computer screen and participants are asked to choose which emotion label best describes the emotion shown. There is no time restriction for answering. Total score ranges from 0 to 60, the separate emotion scores range from 0 to 10The FEEST has shown to have good reliability and validity and has proven to be sensitive in various patient groups, such as acquired brain injury patients [10, 51] and patients with FTD [14].

*The Emotion Recognition Task* (ERT, [46]). Participants are presented with 96 morphed video clips of emotional facial expressions at different intensities. The emotions depicted are anger, disgust, fear, happiness, sadness, and surprise (16 of each). The ERT includes morphs ranging from a neutral expression to four different emotional intensities; 0–40%, 0–60%, 0–80%, and 0–100%. The duration of the morphed video clips ranges from 1 to 3 seconds, after which the static end image maintains on the screen until the participant chooses an emotional label that describes the emotion that is shown. Total score ranges from 0 to 96, the separate emotion scores range from 0 to 16. The ERT has been validated in several neurological and psychiatric patient groups such as, obsessive-compulsive disorder [52], FTD [53], and patients with prefrontal cortex (PFC) lesions [54].

*Shortened Dutch version of The Awareness of Social Inference Test* (TASIT, [27]). TASIT is a social perception measure and consist of three subtests, including the *Emotion Evaluation Test (EET)*. The EET assesses the audiovisual recognition of emotional expressions. Participants are shown 14 videos, in which an actor is engaged in an ambiguous or neutral conversation while portraying one of the six basic emotions (anger, disgust, fear, happiness, sadness, and surprise) or a neutral emotional state, and are asked to select the correct emotion. There are two exemplars of each emotion (and neutral). The duration of the videos ranges from 20 to 41 seconds. Total score ranges from 0 to 12 and the separate emotion scores range from 0 to 2. We did not include the neutral stimuli scores in our study. The original TASIT [47] has been shown to have good reliability, as well as strong validity [24, 55]. Likewise, the Dutch TASIT-short is a valid instrument and has proven to be sensitive to brain injury [27].

#### Neuropsychological functions

*Digit span*. The digit span test is a subtest of the Wechsler Adult Intelligence Scale (WAIS-III) [56] and is a measure of working memory. Participants have to repeat a series of digits both forward and backward. The score is the total amount of correctly repeated series, with a maximum of 30.

*Symbol Digit Modalities Test* (SDMT, [57]). The SDMT consists of a sample line of digits numbered 1 to 9 that are paired with a unique symbol. Participants are presented a sheet containing the unique symbols in random order and the task is to write down, as rapidly as possible, the matching number. The total score (maximum 110) is the number of correct coupled numbers and symbols within 90 seconds and is a measure of attention and information processing speed.

### Statistical analyses

All statistical analyses were conducted using Statistical Package for the Social Sciences (SPSS), Version 23.0. Descriptive statistics were calculated for participant characteristics. Total scores for all three emotion recognition tests were checked for normal distribution and non-parametric alternatives were applied in case of violation of the assumption of normality. Spearman’s correlation coefficients (two-tailed) were used to examine correlations between the emotion subscores and total emotion scores across the three emotion recognition tests. Mann-Whitney U tests were conducted to analyze gender differences on total scores. Furthermore, Spearman’s correlation coefficients (two-tailed) were used to examine the relationship between age, educational level, scores on neuropsychological tests and total scores on the emotion recognition tests. Lastly, Kruskal-Wallis tests were conducted to examine whether there are differences in scores (i.e. practice effects) on the three emotion recognition tests according to protocol version.

Alpha levels were adjusted for multiple comparisons using the Holm-Bonferroni Correction [58].

## Results

Table 1 displays the means, standard deviations and percentages of correct answers of the participants on the FEEST, ERT, and EET (higher scores indicate more accurate emotion identification). The percentage of correct answers was lowest on the ERT.

**Table 1:** Descriptive overview of the mean and standard deviation of the scores on the FEEST, ERT, and EET and the percentage correct answers for separate emotions and total scores per test.

			Emotion Recognition Tests			
	FEEST		ERT		EET	
Measure	M (SD)	% correct	M (SD)	% correct	M (SD)	% correct
**Anger**	7.9 (1.4)	79.2	14.3 (1.8)	89.4	1.6 (0.5)	82.1
**Disgust**	7.9 (2.0)	78.6	10.9 (3.5)	67.6	1.6 (0.5)	82.1
**Fear**	8.1 (2.1)	80.5	7.0 (3.3)	43.5	1.9 (0.2)	97.0
**Happiness**	9.9 (0.4)	98.8	15.1 (1.0)	94.6	1.7 (0.5)	85.1
**Sadness**	7.5 (1.8)	75.2	8.1 (3.9)	50.4	1.7 (0.5)	83.3
**Surprise**	8.9 (1.3)	88.9	9.3 (2.4)	57.9	1.9 (0.4)	92.3
**Total score**	50.1 (5.2)	83.5	64.6 (8.5)	67.2	10.4 (1.4)	87.0

M = Mean; SD = Standard Deviation.

FEEST = Facial Expressions of Emotion Stimuli and Test; ERT = Emotion Recognition Task; EET = Emotion Evaluation Test.

### Correlations between the emotion recognition tests

There were significant positive, but weak, correlations both between the total scores on the ERT and EET and between the total scores on the FEEST and EET (Table 2). A significant moderate, positive correlation was found between the total scores on the FEEST and ERT: a high performance on the FEEST is related to a high performance on both the ERT and the EET.

**Table 2:** Spearman correlations between the total scores and separate emotion scores of the FEEST, ERT, and EET.

Emotions	Emotion recognition tests		
	FEEST and ERT	FEEST and EET	EET and ERT
**Anger**	.24	-.08	.12
**Disgust**	.46*	.05	.19
**Fear**	.36*	.15	.10
**Happiness**	.13	.24	-.20
**Sadness**	.21	.24	.03
**Surprise**	-.06	.08	-.01
**Total score**	.45*	.31*	.35*

*Significant p value < Bonferroni Holm corrected alpha.

FEEST = Facial Expressions of Emotion Stimuli and Test; ERT = Emotion Recognition Task; EET = Emotion Evaluation Test.

Spearman correlations between the separate scores for each of the basic emotions of the three tests are displayed in Table 2. There was a significant weak, positive correlation for the emotion ‘fear’ on the ERT and FEEST as well as a significant moderate, positive correlation for the emotion ‘disgust’. Correlations between the FEEST and EET as well as between the ERT and EET were low and not statistically significant for all separate emotion scores.

### Order effects test battery protocols

**Fig 1:**
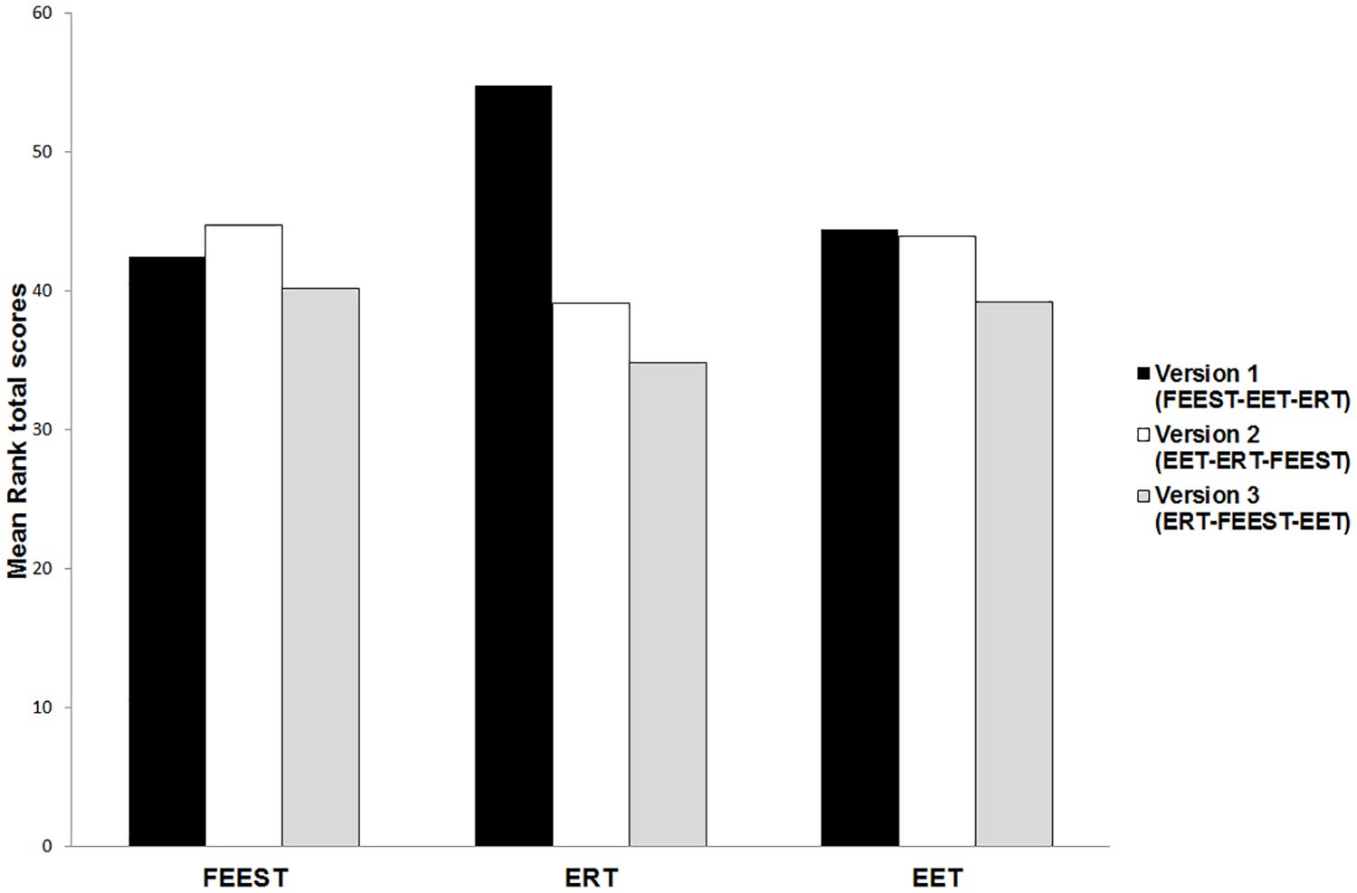
Comparison of the three versions regarding the total emotion recognition scores per test. FEEST = Facial Expressions of Emotion Stimuli and Tests; ERT = Emotion Recognition Task; EET = Emotion Evaluation Test.

Kruskal-Wallis test showed a statistically significant difference in total ERT scores between the three different test battery protocols (X^2^ = 9.99, p = .01), with a mean rank ERT total score of 54.75 for version 1, 39.08 for version 2, and 34.79 for version 3 (Fig 1). There were no significant differences in total FEEST (X^2^ = .51, p = .77) and EET (X^2^ = .81, p = .67) scores found among the three protocols.

### Correlations with demographic variables

In Table 3, Spearman correlations between the total scores on the FEEST, ERT, EET, and the demographic variables age and education are depicted. Lower scores on the ERT seem to correspond with higher age whereas higher educational level seems to be associated with higher scores. No significant correlations were found between age, education and performance on both the FEEST and EET.

**Table 3:** Spearman correlations between FEEST, ERT, EET, demographic variables, and neuropsychological functioning.

Variable	FEEST	ERT	EET
*Demographics*			
**Age**	-.08	-.22*	-.09
**Education**	.17	.29*	.21
*Neuropsychological tests*			
**Digit Span (working memory)**	.26*	.24*	.03
**SDMT (attention, mental speed)**	.27*	-.02	-.01

*Significant p value < Bonferroni Holm corrected alpha.

FEEST = Facial Expressions of Emotion Stimuli and Test; ERT = Emotion Recognition Task; EET = Emotion Evaluation Test; SDMT = Symbol Digit Modalities Test.

A Mann-Whitney U test was conducted to compare the performances of men and women on the three tests for emotion recognition. Results indicated that scores of women were significantly higher than scores of men on all three tests: FEEST (U = 628.00, z = -2.24, p = .03, r = .25, Mdn women = 52, Mdn men = 50); ERT (U = 585.50, z = -2.63, p = .01, r = -.29, Mdn women = 67, Mdn men = 64); EET (U = 619.00, z = -2.37, p = .02, r = .26, Mdn women = 13, Mdn men = 12).

### Correlations with neuropsychological functions

Spearman correlations between the scores on the neuropsychological measures and the total scores on the FEEST, ERT, and EET are presented in Table 3. The results indicate significant weak, positive correlations between the total score on the FEEST and the scores on the Digit Span and SDMT. Additionally, a significant weak, positive correlation was found between the total score on the ERT and the score on the Digit Span test. Thus, higher FEEST and ERT scores were associated with better working memory. Additionally, higher FEEST scores were associated with higher information processing speed. Correlations between the SDMT and ERT as well as between the EET and both the SDMT and Digit Span were low and not statistically significant.

## Discussion

The present study aimed to compare three neuropsychological emotion recognition tests in a group of healthy participants. The type of test stimuli of the three tests differed from static photographs to more dynamic stimuli in the form of morphed photographs to audiovisual displays. The results show significant, moderate correlations between the total scores on the three tests, but only significant correlations between two separate similar emotions (fear and disgust) of the FEEST and ERT. Furthermore, the ERT appeared to have a practice effect; when another emotion recognition test was administered beforehand, participants attained higher total scores. Moreover, the ERT was the only test sensitive to the effects of age and education. Lastly, neuropsychological functions (e.g. mental speed, attention, working memory) were related to the FEEST and not to the dynamic tests as was expected. Our findings indicate that despite some overlap, clear differences exist between the three tests, which suggest that each test measures a unique part of the construct of emotion recognition.

With regard to the comparability of the tests, the highest correlation was found between the ERT and FEEST; correlations of both tests with the EET were lower. This suggest that our previously assumed ranking on a regular continuum from static to more dynamic stimuli, of FEEST to ERT to EET, does not entirely apply, as the ERT seems to have more in common with the FEEST than with the EET. This also may be due to the fact that the greatest difference between the EET and the other two tests is the inclusion of voice in test stimuli. Furthermore, regarding the separate emotions, we found a relationship between the emotions fear and disgust of the FEEST and ERT only. The results did not reveal a relationship between the other same basic emotions; we found no relation between the same basic emotions of the EET and FEEST as well as of the EET and ERT, which indicates that the three tests may not measure the same (basic) emotions.

Since dynamic tests tend to put higher demands on mental speed and working memory [27, 32] an association was expected between the EET and these cognitive functions, but this was not found. A possible explanation might be that the EET is enriched with audiovisual cues which may enhance detection of the correct emotions by enlisting additional emotion processing systems. In contrast, our results did reveal significant correlations between the other two tests and neuropsychological functions. We found a significant correlation between performance on the ERT and working memory. Also, the results revealed a relation between performance on the FEEST and working memory as well as mental speed. This finding is surprising since the FEEST is a static test and it was hypothesized that static test stimuli may activate neuropsychological processes to a lesser extent. However, the nature of the FEEST, where every stimulus is briefly displayed, requires mental speed in order to process all relevant information in time and may draw on neuropsychological processes for that reason.

Regarding the effect of demographic variables on emotion recognition, our results showed no association between both age as well as educational level and performance on the FEEST and EET. Furthermore, in line with previous findings, the current study found that women outperformed men on all three emotion recognition tests [59, 60]. Interestingly, our results demonstrated that performance on the ERT deteriorated with advancing age. Additionally, a significant correlation was found between education and the ERT, indicating that more highly educated participants performed better.

Furthermore, the current study revealed an order effect for one of the three protocols that was used; participants performed better on the ERT when the FEEST was administered beforehand. This possibly displays a practice effect, that is, a change in test performance as a result of increasing familiarity with and exposure to test instruments and/or items [61]. Practice effects can complicate the interpretation of test results and may result in misinterpretation of outcomes and false conclusions [62]. Hence, the ERT seems to be susceptible to practice effects when used in a test battery comprising multiple emotion recognition tests. In addition, participants showed the lowest total scores on the ERT as compared to the other two tests. Based on these results and the correlations between ERT scores and education and age, we cautiously assume that the ERT is more difficult than the FEEST and EET.

Some limitations of this study should be mentioned. Although the level of education in our sample of healthy individuals represents a majority of the Dutch population, we did not include individuals with lower educational levels. This may have limited the generalizability of the results. It is known that education is associated with emotion recognition, so one could therefore argue that a sample consisting of participants with both low and high levels of education may result in more spread of the results. However, the results of two previous studies with samples that also comprised of two lower educational categories, showed comparable standard deviations [51, 63]. Furthermore, the present study only used one subtest (EET) of the Dutch TASIT short, since this subtest is a measure for emotion recognition. However, the scale used for the EET has a very small score range, which may have influenced the differences in emotion recognition performance between the three tests as seen in the results. A score of 100% correctly recognized emotions can be achieved faster; however, same is true for achieving a zero score. Furthermore, since we made use of the shortened version of TASIT which has a restricted score range, it might have reduced the likelihood of seeing correlations with other measures. It is conceivable that the correlations would have turned out slightly stronger when the original version of the test was used. Thus, although the EET is a measure of emotion recognition, it does not seem to be a reliable instrument when used as a separate test. In clinical settings, it would be reasonable to administer all subtest of the Dutch TASIT short.

In conclusion, the present study shows that three different emotion recognition tests, with either static or dynamic stimuli, each measure a unique part of the construct. In our sample of healthy participants, performance on the ERT was lowest when compared to the other two tests. Besides, this test shows to be sensitive to the effects of age and education and seems to be susceptible for practice effects when participants were exposed to other emotion recognition tests. One could therefore argue that the ERT may be more difficult in comparison to the other two tests, which might lead to problems in interpreting the results in clinical settings and highlights the importance of using norms. Lastly, our results show that there exists an association between neuropsychological functions and the FEEST, a test with static stimuli. This association was found in a lesser extent for the ERT, and not found for the EET, which are both tests comprising of dynamic test stimuli.

The results of our study are of importance for clinical practice. Deficits in emotion recognition have been found to have great negative consequences for health and mental well-being [64, 65]. It is well known that neurologic patient groups often show impairments in the ability to accurately recognize facial expressions [51, 66, 67]. Therefore, in clinical (rehabilitation) settings, adequate assessment of emotion recognition is crucial to measure deficits and predict social problems in everyday life. Future research may focus on the examination of both dynamic and static emotion recognition tests in various patient groups to provide further evidence of the utility of these tests in clinical settings.
